# Corrosion of High-Strength Steel Wires under Tensile Stress

**DOI:** 10.3390/ma13214790

**Published:** 2020-10-27

**Authors:** Shanglin Lv, Kefei Li, Jie Chen, Xiaobin Li

**Affiliations:** 1 Department of Civil Engineering, Tsinghua University, Beijing 100084, China; lvshanglin@126.com; 2 National Construction Steel Quality Supervision and Test Centre, Central Research Institute of Building and Construction, China Metallurgical Group Cooperation, Co., Ltd., Beijing 100088, China; xjj_xjj@163.com (J.C.); lxb2001230@126.com (X.L.)

**Keywords:** high-strength wires, stress corrosion cracking, potentiodynamic polarization, electrochemical impedance spectroscopy, metallography, fracture morphology

## Abstract

The stress corrosion cracking is the central issue for high-strength wires under high tensile stress used in civil engineering. This paper explores the resistance of stress corrosion cracking of three typical steel wires of high-strength carbon through a laboratory test, combining the actions of tensile stress and corrosive solution. Besides, the impact of tensile stress and immersion time are also investigated. During the tests, the wires were subject to electrochemical measurements of potentiodynamic polarization and electrochemical impedance spectroscopy, and the microstructure analysis was performed on the fractured cross sections. The obtained results show the following: the high-strength wire, conforming to GB/T 5224, has higher resistance to the combined actions of tensile stress and corrosive solution; tensile stress of 70% fracture strength and longer loading-immersion time make the film of corrosion products on steel surface unstable and weaken the corrosion resistance; the surface film consisted of the iron oxide film and the corrosion products film whose components are mainly iron thiocyanate and iron sulphide.

## 1. Introduction

Due to the high bearing capacity and stiffness, the prestressed concrete (PC) structures are widely used in large-span building, bridge and hydraulic structures [1]. The PC structures adopt either bonded or unbounded systems and are usually considered to acquire sufficient durability due to the limitation of concrete cracking and appropriate protection measures for the high-strength wires and strands [2,3]. However, prestress failures in PC structures have been reported in literature. Schupack and Suarez [4] showed, through an investigation from 1978 to 1982 in USA, about 50 PC structures had undergone different degrees of corrosion, among which 10 cases were caused by stress corrosion or hydrogen brittleness. Hydrogen is introduced by the improperly pickling process in general. As for the hydrogen-induced stress corrosion cracking mechanism, Vehovar et al. [5] reported its cause as the embrittlement of the prestressing tendons due to the penetration of hydrogen atoms, generated by hydrogen reduction at the alloy’s surface. Enos and Scully et al. [6] used a simulated steel-concrete interface and laboratory-scale prestressed concrete pilings to study the safe cathodic protection limits for prestressing steel in concrete about hydrogen embrittlement. Moreover, the dislocations in the prestressing tendons relative to the production process and heat treatment are sensitive to hydrogen embrittlement. On this basis, Gertsman [7] and Jaka Kovac et al. [8] studied and characterized the intergranular SCC (IGSCC) processes. The results show that geometrical parameters and chemical parameters play an important role in the intergranular stress corrosion cracking of materials.

Walter [9] reported 242 cases of prestress failure, from 1951 to 1979, among which a large portion were attributed to the environmental actions. Among these failure causes, the stress corrosion cracking (SCC) is always a main engineering concern as it reduces the fracture strength of high-strength steel wires, leading to unexpected brittle failures of PC structures [10,11]. Moreover, the SCC can be sensitive to the aggressive agents present in the corrosive environments [12].

The SCC is caused by the combined action of stress, environmental agents and metal components, and the widely accepted mechanisms include the surface (depassivation) film of wires is broken jointly by the tensile stress and the environmental actions; the freshly exposed steel surface serves as the anode dissolved at the crack tip, leading to the steel fracture [13,14]. The conventional test methods include the deformation, sustained loading and slow strain rate testing [15]. During the mechanical loading, the electrochemical methods have been used to characterize the SCC process, identifying the susceptibility potential range for prestress wires [8,16] and detecting the stress corrosion fracture of a stainless steel through the phase shift of electrochemical impedance spectroscopy (EIS) [17]. As for the corrosive environment, a SCC test method was developed by the International Prestressed Concrete Federation (FIP, now merged into *fib*), using ammonium thiocyanate (NH_4_SCN) solution as the corrosive environment for SCC test. This method has been standardized by ISO 15630 [18] and GB/T 21839 [19]. Perrin et al. [20] used this method to investigate the SCC mechanisms together with detection of EIS, electrochemical noise and metallographic analysis, and confirmed the validity of this method for SCC investigation.

Nowadays, high-strength steel wires for civil engineering use are usually classified following the mechanical properties including the rupture strength and the elongation, with a rough account for chemical compositions [21,22,23]. However, the SCC risk of high-strength steel is rather sensitive to the chemical composition. Thus, the SCC performance should always be investigated with respect to the environmental actions and steel chemical compositions. Accordingly, it is of interest to compare the SCC performance for streel wires with similar chemical compositions from different standards. To this purpose, this study retains three steel wires, conforming to BS 5896 [21] and GB/T 5224 [22], and investigates the SCC resistance of these wires through stress-immersion tests and the role of stress on the electrochemical behaviors.

## 2. Experiments

### 2.1. Material and Composition

The samples used in this investigation were central wires of prestress strand. Three kinds of samples were chosen: Sample A from Jiangyin, China, Walsin steel cable Co., Ltd., conforming to BS 5896 [21] and using C86D2 grade steel [24] and Samples B and C, according to GB/T 5224 [22] and using YL87B grade steel [25] but from different manufacturers (Xinhua Metal Products Co., Ltd., Xinyu, China and, Walsin steel cable Co., Ltd., Jiangyin, China respectively). The chemical compositions of steel samples were determined by GB/T 4336 [26], and the results are given in Table 1. Besides, the measurement uncertainty for chemical composition is given in Table 2.

### 2.2. Specimen Preparation

The rupture strength *f*_t_ of wires, A, B and C, were measured through axial tension loading tests on three specimens for each wire in lab-air environment, and the average values were retained as its fracture strength: 1971 MPa, 2106 MPa and 2088 MPa for Wires A, B and C, respectively. The relative composite measurement uncertainty for fracture strength values 1971 MPa, 2106 MPa and 2088 MPa is 0.699%, 0.722% and 0.718% respectively.

**Figure 1:**
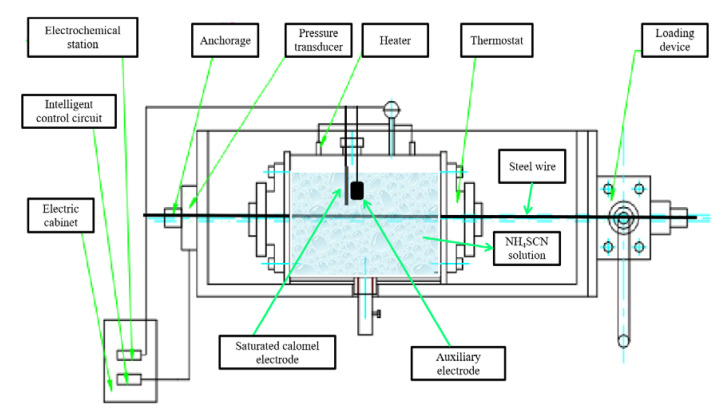
Experimental setup for accelerated corrosion tests for prestressed wires.

For the immersion tests, the sample length is 1280 mm, twice as long as the immersion part in the solution, and the diameter of sample is 5 mm. The NH_4_SCN solution was prepared by dissolving 200 g of analytically pure NH_4_SCN in 800 mL of distilled water. The stress corrosion testing device is composed of a loading frame and an immersion pool; see Figure 1. During the immersion tests, electrochemical measurements were performed for the steel wires in immersion.

### 2.3. Test Procedure

The samples were wiped, degreased with acetone (CH_3_COCH_3_), and air-dried. At least 50 mm in length parts at both ends of the samples were coated with sealant to prevent corrosion. The samples were loaded to 70% of the rupture strength and the loading was sustained, with variation controlled within +2%, during the entire test duration. After loading, the container was sealed to prevent leakage, and the solution was replaced after each test. The NH_4_SCN solution (Tengtai Chemical Technology Co., Ltd., Suzhou, China) was first deoxidized by introducing nitrogen gas during 2 h, then pre-heated to 50–55 °C, and injected into the container and kept at a constant temperature. The solution was filled within 1 min, covering the surface of sample and kept stagnant during the stress corrosion tests. The following cases were tested: (1) Samples A, B and C under stress level 70% *f*_t_ with loading-immersion duration 10 min; (2) Samples A under free stress (0% *f*_t_) with loading-immersion duration 10 min, and stress level (70% *f*_t_) with loading-immersion duration 30 min.

During all these tests, the potentiodynamic polarization and EIS (Chenhua Instrument Co., Ltd., Chi600E, Shanghai, China) were used to characterize the corrosion behavior of steel wires. The electrochemical measurements were performed via a three-electrode system containing a stainless-steel auxiliary electrode and a saturated calomel electrode (SCE) as reference, and the potentials hereafter refer to the SCE. The prestressed steel wire, acting as working electrode, was immersed in NH_4_SCN solution with an exposed working area of 100 cm^2^. The electrochemical tests were conducted when the open circuit potential (OCP) of the working electrode became stable. The scanning potential of the anodic polarization curve was −1.5–1.0 V vs. OCP, and the scanning rate was 5 mV/s. The sinusoidal voltage excitation signal with disturbance amplitude of 5 mV vs. OCP was used for EIS testing, and the frequency range was 10^5^–10^−2^ Hz. All the electrochemical measurements were repeated three times for good reproducibility, and a typical group of data were selected to study for clarity.

## 3. Electrochemical Analysis

### 3.1. Potentiodynamic Polarization Analysis

**Figure 2:**
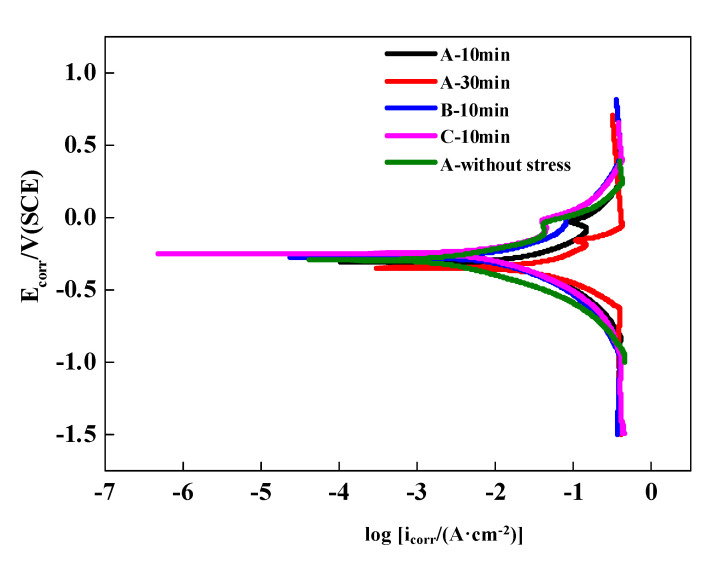
Potentiodynamic polarization curves of steel wires in NH_4_SCN solution.

The potentiodynamic polarization results are given in Figure 2 and Table 3. For the cases of loading-immersion duration of 10 min, the potentiodynamic polarization curves of wires B and C were on the left side of Wire A, and the curves of B and C were rather close. For the case of Wire A of loading-immersion duration 30 min, the polarization curve of wire A moves towards the bottom right, and the stable range becomes narrower, indicating that longer exposure time promotes the anodic dissolution of steel and the corrosion resistance is weakened [27,28]. Furthermore, compared to the A—10 min case, the case of A-without stress presents a left-upward shift in the polarization curves, showing wider range of the stable interval and the degree of corrosion was minimized [29]. 

In Table 3, the corrosion potential (*E*_corr_), corrosion current density (*i*_corr_), anodic Tafel slope (*b*_a_) and cathodic Tafel slope (*b*_c_) values were taken from the polarization curves in Figure 2. Compared to the cases B, C—10 min, the case A—10 min has lower *E*_corr_, *b*_a_ and *b*_c_ values but larger *i*_corr_ values. By extending the loading-immersion time from 10 min to 30 min (Case A—10 min to Case A—30 min), the value of *i*_corr_ increased from 1.28 × 10^−2^ A/cm^2^ to 1.90 × 10^−2^ A/cm^2^, due to the *E*_corr_ descended from −315.5 to −349.0 mV vs. SCE. Compared to Case A—10 min, the stress-free case, A-without stress, has the *E*_corr_ value increased from −315.5 mV to −290.3 mV and *i*_corr_ decreased from 1.28 × 10^−2^ A/cm^2^ to 0.34 × 10^−2^ A/cm^2^. From these values, the current density of A was higher than those of B and C under the same corrosion conditions, which indicates that wires B and C had lower corrosion rate via the immersion tests. Moreover, for wires A, the current density increased with the application of tensile stress loading and longer loading-immersion duration, indicating that the corrosion process was accelerated. Tang [30] studied the effect of stress on corrosion of *X*70 pipeline steel in neutral solution with microzone electrochemical method. They found that the corrosion rate increased significantly with the stress loading, which confirmed that stress promoted the occurrence of corrosion. 

### 3.2. EIS Analysis

**Figure 3:**
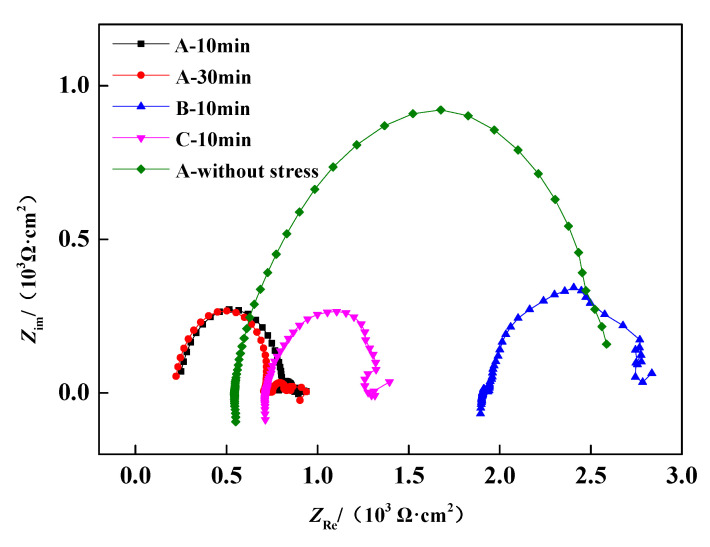
Nyquist curves of steel wires of different cases in NH_4_SCN solution.

Figure 3 provides the experimentally obtained Nyquist curves and the EIS of response evolution of the corrosion products in different cases. Nyquist curves for the prestressed wire samples showed the similar characteristics. All of the Nyquist curves showed incomplete depressed semi-circular arcs that were affected by the frequency dispersion, and inductive shrinking occurred in the low-frequency zone, showing that such adsorbents as ferro-thiocyanate film existed on the steel surface and the corrosion was activated during this frequency range [26]. Under the same stress-loading duration (A, B, C—10 min cases), the Nyquist curves showed that the capacitance arc magnitude of B were larger than those of A and C, which suggests a more compact film of corrosion products was formed on the surface of B and the corrosion resistance of B wire is better than A and C wires. The polarization curves are extrapolated from the dynamic electrode behavior of strong polarization, while the EIS is measured under the stable equilibrium state. Therefore, the behavior of the electrode in static equilibrium can be better characterized by EIS results.

Further, as the loading-immersion time increases, from 10 min (A—10 min) to 30 min (A—30 min), the capacitance arc magnitudes are basically the same. By comparing the Nyquist curves of stressed wires (A, B, C under 70% *f*_t_) and stress free wire (A, 0% *f*_t_), it was found that the capacitance arc magnitude of stress free wire A is much larger than the wires (A, B, C) under 70% *f*_t_ tensile stress, indicating that the tensile stress promotes substantially the occurrence of corrosion. This observation is consistent with the anodic polarization curves in Figure 2. In Figure 3, the *Z*_re_ values, real part of the impedance, were not the same for different wires. This is due to the fact that the NH_4_SCN solution was replaced and refilled into the accelerated corrosion container after each individual wire test, resulting in a slight change in the corrosive environment. However, this change in *Z*_re_ value does not change the judgement on the role of tensile stress.

**Figure 4:**
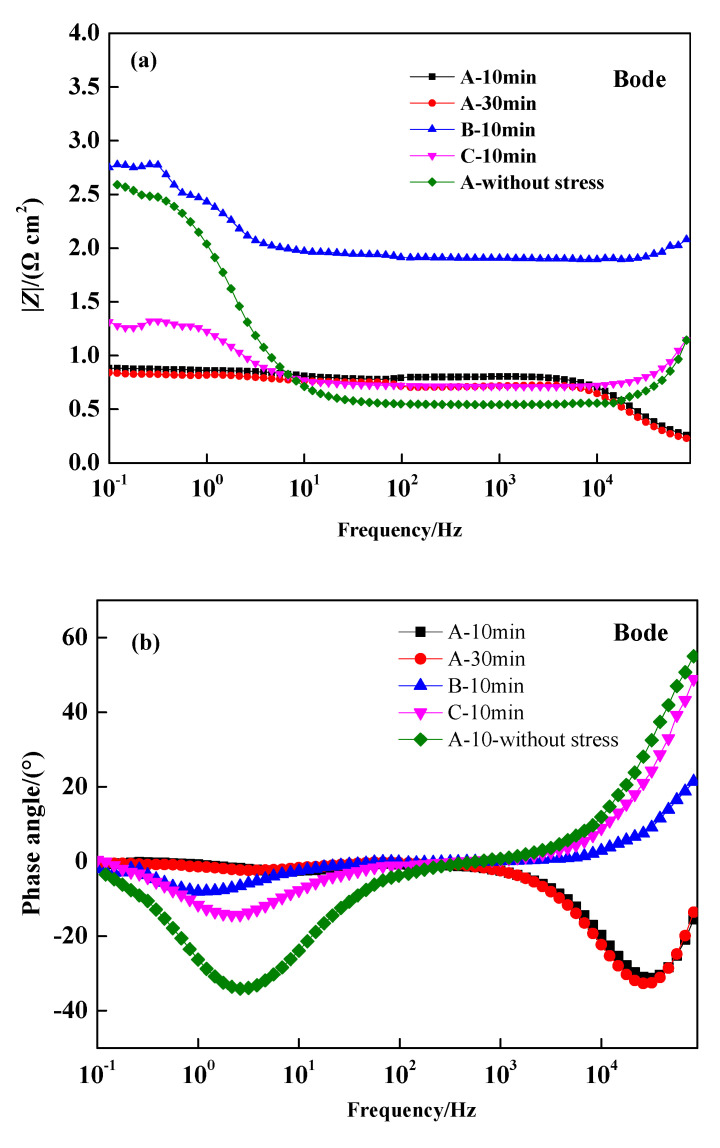
Bode curves of steel wires immersed in NH_4_SCN solution: impedance modulus (**a**) and impedance angle (**b**).

The polarization resistance can be obtained from low frequency impedance, which determines the change in transfer resistance [31]. As shown in Figure 4a, when the influence caused by the change of solution impedance was ignored, the Bode curves of different wires revealed that the impedance modulus of Wire C in the low frequency region was slightly higher than that of Wire A under the same loading-immersion duration. Moreover, the impedance modulus of B was much higher than those of A and C, which indicates that the surface film resistance of B was higher and a significant charge transfer resistance of Wire B was created; therefore, the corrosion resistance of Wire B was higher than Wires A and C. Figure 4b shows the phase angle of three stressed wires (A, B, C—10 min) increased with the frequency under the same stress-corrosion time. The peak value of C was the highest, showing that the surface was smoother, and the pitting corrosion was inhibited. With corrosion time prolonged from 10 min to 30 min, the impedance modulus and phase angle of Wire A decreased, and the corrosion resistance decreased accordingly. When Wire A is under stress-free condition, the resistance of the surface film in the low frequency region was larger than the stressed case of A—10 min, and the resistance decreased also with frequency. Meanwhile, the peak value of stressed A—10 min was lower than the stress-free case, indicating that the formation of compact products was inhibited under tensile stress, and the product film was rougher. 

**Figure 5:**
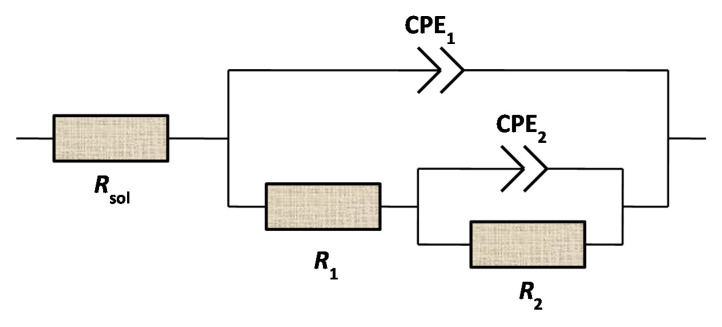
Equivalent electrical circuit used to represent the measured EIS data.

The models in literature [32,33,34] are used to describe the electrochemical processes of the surface films exposed to different conditions, and interpret the obtained EIS curves, see Figure 5. In the figure, *R*_sol_ represents the resistance of the solution, the constant phase angle element CPE_1_ corresponds to the double-layer capacitance of the interface between the sample surface and solution, *R*_1_ represented the charge transfer resistance, *R*_2_ was the surface film resistance, the constant phase angle element CPE2 was used as a substitute for the surface film capacitance, and *n* represented the dispersion exponent. Through this model, the electrochemical parameters can be regressed to represent corrosion kinetics for different cases. In general, the electron transfer resistance determines the impedance at low frequencies, the solution resistance determines the impedance at high frequencies, and the electrochemical corrosion kinetics can be extrapolated by the low frequency impedance.

Due to the heterogeneity of the surface film [35], a constant phase element (CPE) is used to represent the non-ideal capacitance responses of the interface. The CPE was defined as,

(1) $$Z_{\mathrm{CPE}}=\frac{1}{Y_{0}{\left(jw\right)}^{n}}$$

where *Y*_0_ is the admittance magnitude of CPE; *ω* is the angular frequency; *j* is the imaginary number (*j*^2^ = −1) and *n* is the exponent (−1 < *n* < 1). *Y*_0_ and *n* can be converted into CPE. The *n* value is interrelated to the heterogeneity and smoothness of the surfaces. When *n* values are close to 1, the CPE will approach an ideal capacitance.

The parameters are regressed and given in Table 4 from the EIS data for steel wires in different cases. Under the same stress-corrosion time (70% *f*_t_, 10 min), the *R*_1_ value of Wires B and C are higher than Wire A, indicating the transfer of electrons is more difficult in Wire B and C. Therefore, Wires B and C show better resistance to the redox reaction of corrosion relative to A. The *R*_2_ value of Wire B is the highest among A, B, C—10 min cases, which means that Wire B has the largest surface film impedance and highest compactness. Compared to Wires B and C, the CPE_1_ and CPE_2_ values of Wire A are slightly higher, showing Wire A is more prone to corrode. The values from A—10 min and A—30 min cases show a general decrease of resistance and capacitance values, indicating the charge transfer processes were promoted with the immersion time from 10 min to 30 min.

The *R*_1_ values of Sample A cases showed the longer loading-immersion duration, in immersion solution, tends to decrease R_1_ value, in other terms, promote the electron transfer and weaken the corrosion resistance. The *R*_2_ values decreased accordingly by longer loading-immersion duration and tensile stress, possibly attributed to the deterioration of surface film. The evolution of CPE_1_ and CPE_2_ increased with prolonged loading-immersion time, indicating that the surface film formed under prolonged loading duration leads to capacitive behaviors, and the corrosion resistance is deteriorated. In addition, with the increase in loading-immersion duration, *n* (*n*_1_, *n*_2_) values of the double layer capacitance and the surface film capacitance decreased, respectively. These observations confirm that the corrosion of steel wires was gradually intensified. Finally, when comparing the stress-free case of Wire A with other stressed cases, one gets a similar *R*_sol_ value but a much higher *R*_1_ value in Table 4, meaning the electrons transfer resistance in stress-free wire A is much higher than those stressed wires (A, B or C). When pitting corrosion occurred on the surface of the stressed wire, the potential of pitting corrosion area was lower than that of other parts, which resulted in the area that became active and provided crack core for stress corrosion. The concentrated stress made the crack tip and the surrounding area yield deformation, and then the micro-slip destroyed the surface film of the crack tip again, which accelerated the dissolution of the tip. At the same time, because of the existence of micro cracks, the corrosion resistance of the surface film deteriorated, and the electron transfer resistance decreased. This observation confirms further that the tensile stress, 70% *f_t_*, substantially decreased the surface electron transfer resistance, thus promoting the SCC of the wires.

## 4. Microstructure Analysis

### 4.1. Metallography

**Figure 6:**
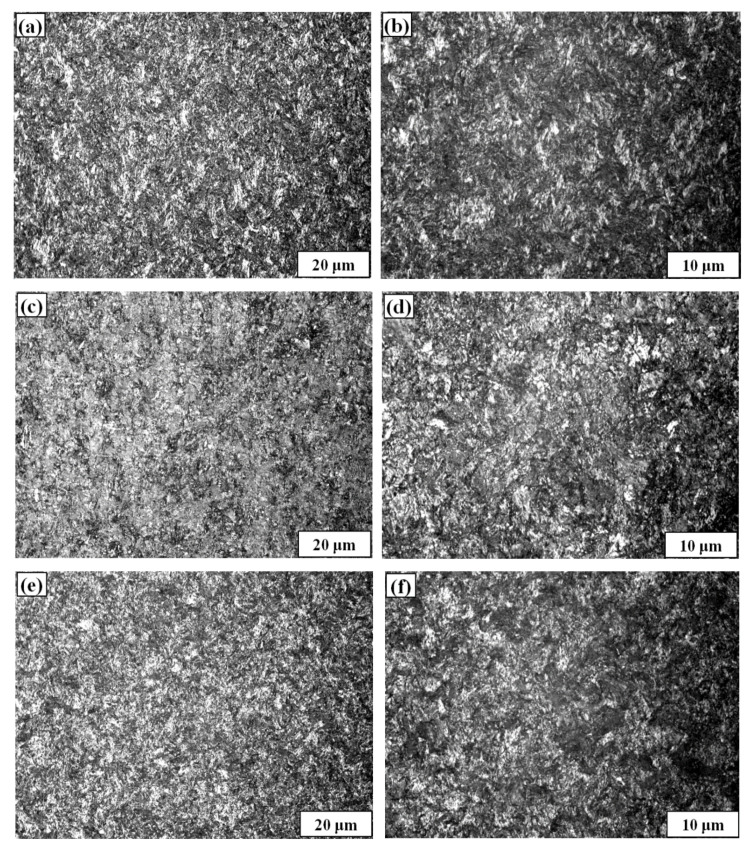
Metallographic microstructures of three high strength wires: Wire A ×500 (**a**) and ×1000 (**b**), Wire B ×500 (**c**) and ×1000 (**d**), Wire C ×500 (**e**) and ×1000 (**f**).

The microstructure of high strength wires was observed by metallography microscope (Olympus GX51, Tokyo, Japan) (Magnification factor: 50×–1000×, Light source: 6V30WHAL halogen lamp) before the corrosion test. Figure 6 shows the metallographic microstructures of Wires A, B and C: All the three wires are composed of sorbite, product of austenite isothermal transformation, having eutectoid structures of alternate thin layers of ferrite and cementite. The sorbite lamellae size of B and C is finer; the grain boundary defects are accordingly reduced, and the corrosion resistance is expected to enhance. The results are consistent with Ren’s study [36].

The ferrite and cementite were grey-white in Figure 6, and the interface between them was black. The three wires all had sorbitization treatment applied to ensure a high degree of strength and toughness. When the wires are produced, chemical composition adjusting is often used to reduce the lower limit temperature of sorbite transformation and avoid the occurrence of bainite and martensite. Generally, the alloying elements Mn, Cr, Ni and Cu will improve the stability of austenite and delay the decomposition of austenite. As shown in Table 1, the carbon content of Samples A, B and C is similar while the Mn, Cr and Cu contents of Wires B and C are higher. Therefore, the completion of the sorbite transformation is prolonged, the precipitation of eutectoid ferrite and pearlite is reduced, and the termination temperature of the sorbite transformation is also significantly decreased; all these factors give Wires B and C higher tensile strength. In addition, the surface film of wires B and C formed by the corrosion was more compact due to the higher content of Cr and Ni. The corrosion resistance was improved, which was consistent with the anodic polarization curve and EIS conclusion.

### 4.2. Fracture Surface

**Figure 7:**
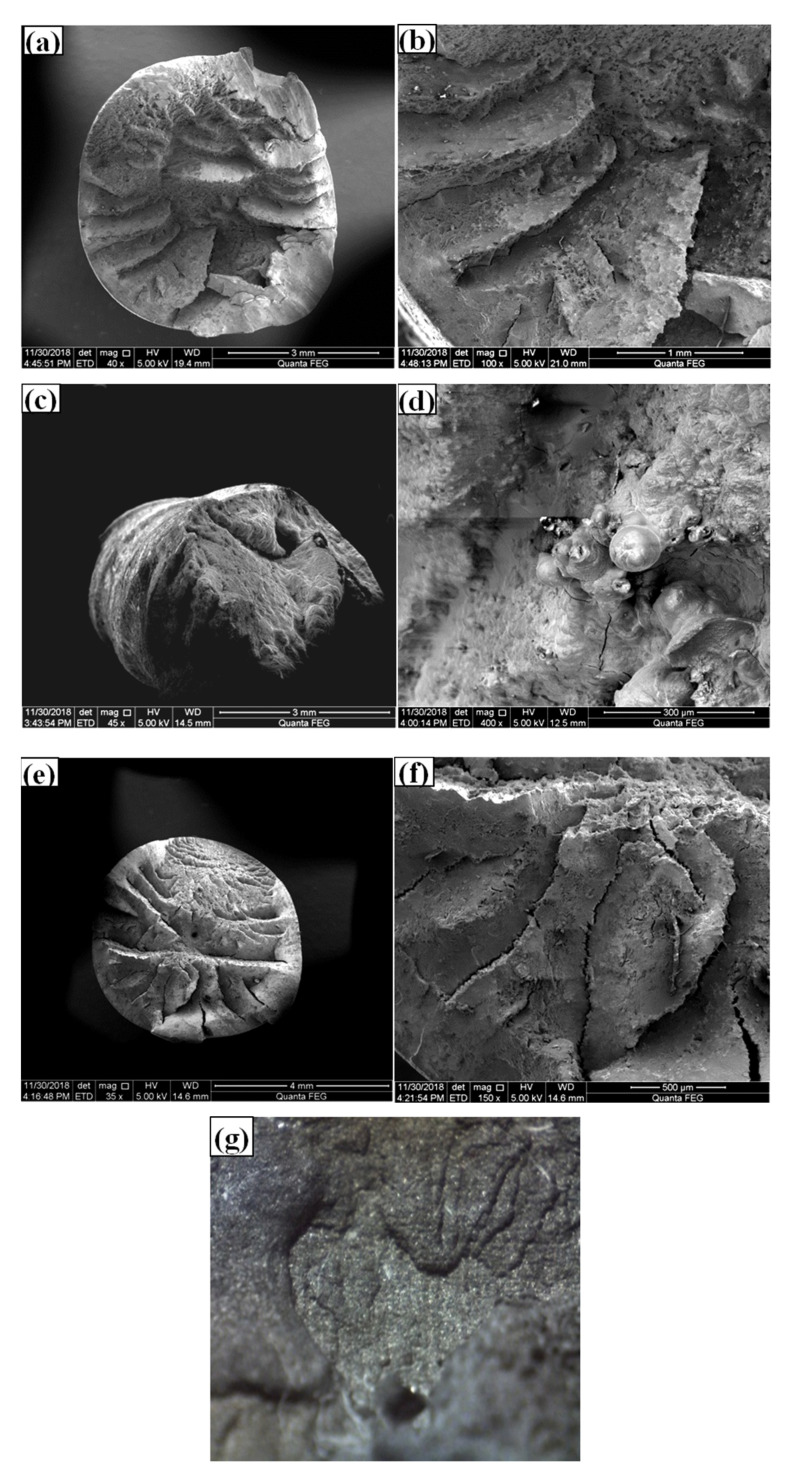
SEM images of rupture surface of wires: (**a**,**b**) wire A, (**c**,**d**) wire B, (**e**,**f**) wire C, (**g**) corrosion pits.

The fracture cross sections of the three wires (A, B, C—10 min) under 70% *f_t_* tensile stress were observed by Scanning Electronic Microscopy (SEM, Quanta 250 FEG, Hillsboro, OR, USA). The acceleration voltage was 20 kV, and high vacuum mode was <6 × 10^−4^ Pa, resolution was 1.2 nm; see Figure 7. 

The fractographic and SEM images in Figure 7 confirmed that the macroscopic fracture of all three wires under stress corrosion is of brittle nature. These fracture surfaces present radial pattern without plastic deformation. The splitting surface is strip-like, and the end of the fracture is radial from the section center to the periphery, which was consistent with the literature observations [37,38]. Under higher magnifications, all sections showed more characteristics related to corrosion: the corrosion pits on the sections are generally deep and narrow, and the surface fractures develop into the solid matrix of steel. These fractures were developed from the microcracks on the wire surface extended along the wire length and tension, results of tensile stress and NH_4_SCN solution. Some cracks, torn edges and quasi-cleavage fractures are found on the wire surface in the crack growth area. The fractography analysis of these fractures attributes the major pattern to be trans-granular.

**Figure 8:**
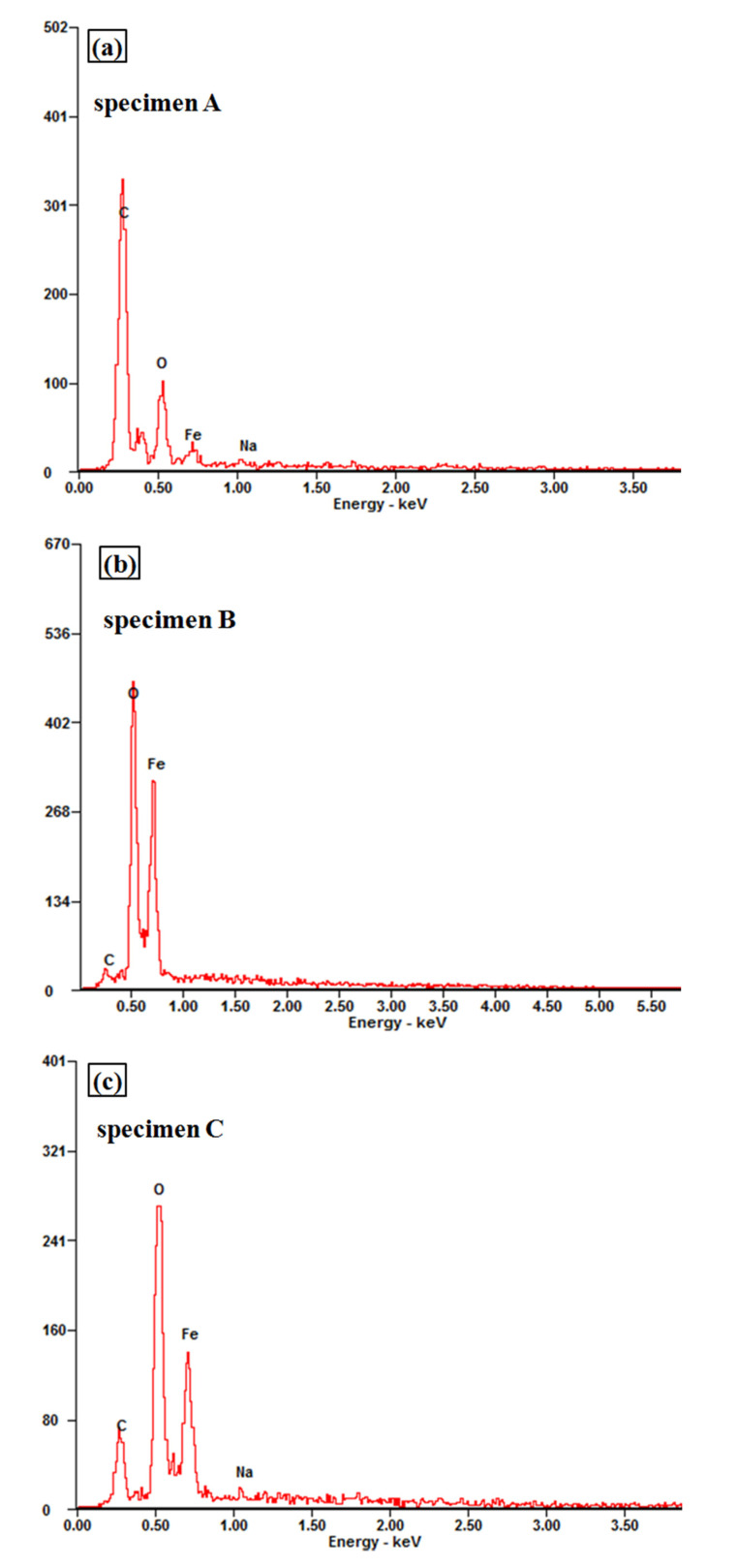
EDX analysis of the fracture inclusions on cross sections of high-strength wires: (**a**) A, (**b**) B and (**c**) C.

Energy spectrum (EDX) was used to analyze the chemical composition of inclusions in the fractures in Figure 8: Wire A has carbides non-metallic inclusions while Wires B and C had oxide non-metallic inclusions. The existence of non-metallic inclusions in the samples destroyed the continuity of the metal matrix structure, which deteriorates the mechanical properties of all the wires. These inclusions can be the crack nucleation sites, which promotes the fracture propagation in solid matrix of granular nature, especially under the high level of tensile stress.

### 4.3. Corrosion Products

The original oxide film defects of steel wire were split to expose the fresh metal under tensile loading. The exposed substrate and the oxide film around the defects formed a corrosion source in NH_4_SCN solution. The exposed fresh metal acted as anode while oxide film acted as cathode, which led to an increase in the corrosion rate. The corrosion products film is formed in the anodic dissolution process, and the defects trigger continuously under tensile loading leading the film to be deteriorated. 

Some ferric iron formed in the anodic dissolution process, and the reaction of ferric iron in solution containing thiocyanate produces ferro-thiocyanate compound, i.e., Fe^3+^ + xSCN^−^→[Fe(SCN)_x_]^3−x^. The ferro-thiocyanate compounds form on the steel surface and will promote the anodic dissolution of steel [30]. From the available EIS and other results in this study, the film becomes loose, and the surface of the steel wire becomes rougher under tensile loading. The decreased transfer resistance and the sharp increase of corrosion current under tensile stress indicate that the film was rapidly deteriorated, which is confirmed also by potentiodynamic polarization curves. 

**Figure 9:**
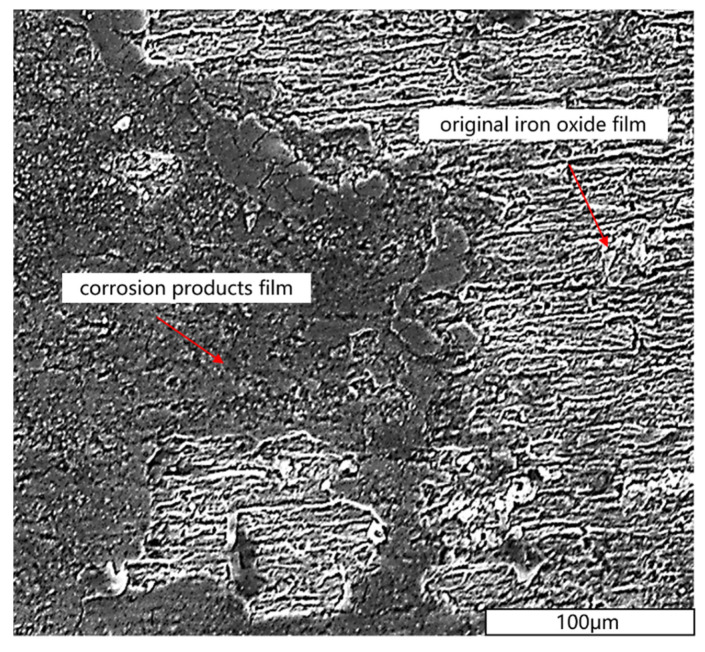
SEM microstructure of prestressed steel wire surface in NH_4_SCN solution.

**Figure 10:**
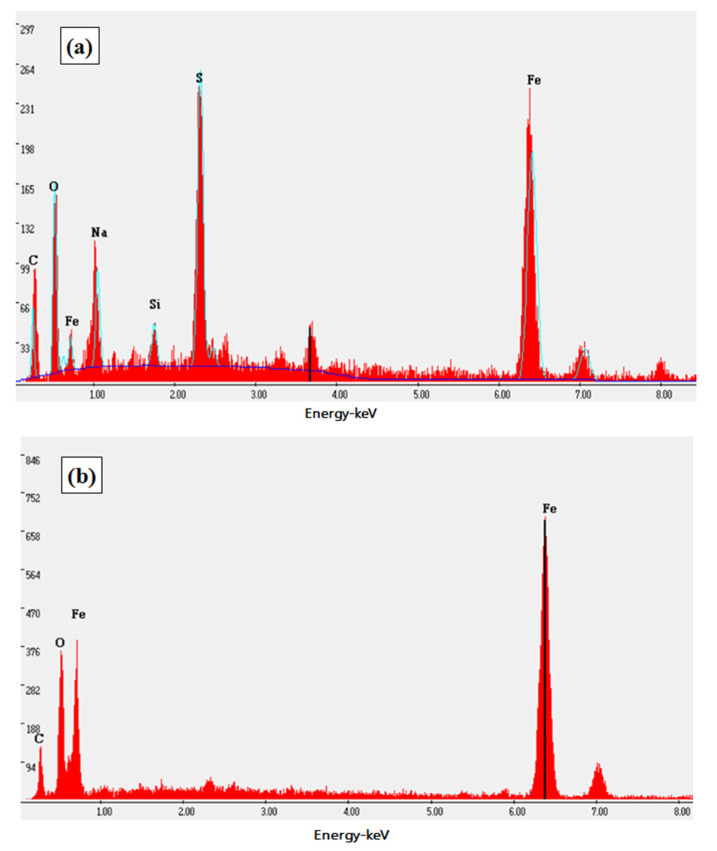
Energy spectrum of prestressed steel wire surface in NH_4_SCN solution: (**a**) corrosion products film, (**b**) original iron oxide film.

Figure 9 and Figure 10 showed the SEM microstructure and the energy spectrum analysis of prestressed steel wire surface after corrosion in NH_4_SCN solution, respectively. The surface film consisted of the original iron oxide film and the corrosion products film whose components are mainly iron thiocyanate and iron sulphide. The original iron oxide film was dense while the corrosion products film was rough and loose. 

The steel wires with similar chemical composition were selected to study the stress corrosion behavior in this paper. The results showed that they had different corrosion resistance in the same corrosion environment. It indicated that even if the contents of Cr, Ni and Cu increased slightly, the performance of corrosion product film on the wires surface would be affected. Meanwhile, stress corrosion is the result of multiple factors synergy, such as composition and microstructure. Thus, in order to improve the stress corrosion resistance of steel, it is necessary to improve many aspects.

## 5. Conclusions

1. The electrochemical measurements show that, with loading duration, the corrosion current density increases, and the corrosion resistance is weakened. The results from polarization curves and EIS are consistent with those from corrosion morphologies.
2. The tensile stress leads to a more unstable corrosion products film formed on the steel, which means worse flatness and compactness. The stress substantially decreased the surface electron transfer resistance, thus promoting the corrosion of the high strength wires.
3. The surface film consisted of the original iron oxide film and the corrosion products film, whose components are mainly iron thiocyanate and iron sulphide.
4. The corrosion-resistance performance of the wires, conforming to GB/T 5224, is better than the selected wire conforming to BS 5896.
5. The corrosion resistance of steel wire will be affected by the change of chemical composition. Stress corrosion is the result of multiple factors synergy, such as composition and microstructure.

## Figures and Tables

**Table 1:** Chemical composition of steel wires (mass fraction)/%.

Wire	C	Si	Mn	P	S	Cr	Ni	Cu	V
A	0.870	0.235	0.694	0.0184	0.0121	0.007	0.009	0.012	0.007
B	0.875	0.253	0.795	0.0188	0.0113	0.190	0.010	0.024	0.005
C	0.886	0.267	0.837	0.0202	0.0109	0.280	0.014	0.031	0.004

**Table 2:** Measurement uncertainty for chemical composition (%).

Wire	C	Si	Mn	P	S	Cr	Ni	Cu	V
A	0.0229	0.0116	0.0167	0.0017	0.0016	0.0031	0.0012	0.0015	0.0052
B	0.0230	0.0121	0.0185	0.0017	0.0015	0.0069	0.0013	0.0024	0.0089
C	0.0233	0.0125	0.0194	0.0018	0.0016	0.0087	0.0016	0.0028	0.0107

**Table 3:** Electrochemical parameters of polarization curves of prestressed steel wires.

Steel Wire	Cases	*E*_corr_	*i*_corr_	*b*_a_	*b*_c_
		(mV vs. SCE)	(10^−^^2^ A/cm^2^)	(V/dec)	(V/dec)
A	70% *f*_t_, 10 min	−315.5	1.28	25.76	13.45
A	70% *f*_t_, 30 min	−349.0	1.90	12.13	9.55
A	0% *f*_t_, 10 min	−290.3	0.34	85.31	23.34
B	70% *f*_t_, 10 min	−286.1	0.65	64.13	15.63
C	70% *f*_t_, 10 min	−281.2	0.51	58.46	18.03

**Table 4:** Fitting parameters for experimental EIS of wires immersed in NH_4_SCN solution.

Steel Wire	Cases	*R*_sol_ (10^3^ Ω cm^2^)	*R*_1_ (10^3^ Ω cm^2^)	CPE_1_		*R*_2_ (10^3^ Ω cm^2^)	CPE_2_	
				*Y*_0_/(10^−4^ Ω^−1^ cm^2^ S^n^)	*n*_1_		*Y*_0_/(10^−4^ Ω^−1^ cm^2^ S^n^)	*n*_2_
A	70% *f*_t_, 10 min	0.26	0.32	1.035	0.95	0.47	1.29	0.91
A	70% *f*_t_, 30 min	0.24	0.29	0.63	0.82	0.42	1.58	0.84
A	0% *f*_t_, 10 min	0.53	1.23	0.11	0.97	0.82	1.19	0.95
B	70% *f*_t_, 10 min	1.86	0.46	0.29	0.96	0.58	1.14	0.93
C	70% *f*_t_, 10 min	0.67	0.42	0.23	0.95	0.52	1.08	0.92
